# Characteristics of Myocardial Postsystolic Shortening in Patients with Symptomatic Hypertrophic Obstructive Cardiomyopathy before and Half a Year after Alcohol Septal Ablation Assessed by Speckle Tracking Echocardiography

**DOI:** 10.1371/journal.pone.0099014

**Published:** 2014-06-12

**Authors:** Jiansong Yuan, Shi Chen, Shubin Qiao, Fujian Duan, Jiafen Zhang, Hao Wang

**Affiliations:** 1 Department of Cardiology, State Key Laboratory of Cardiovascular Disease, Fuwai Hospital, National Center for Cardiovascular Disease, Peking Union Medical College and Chinese Academy of Medical Sciences, Beijing, China; 2 Department of Cardiology, West China Hospital, Sichuan University, Chengdu, Sichuan, China; 3 Department of Echocardiography, State Key Laboratory of Cardiovascular Disease, Fuwai Hospital, National Center for Cardiovascular Disease, Peking Union Medical College and Chinese Academy of Medical Sciences, Beijing, China; University of Louisville, United States of America

## Abstract

**Objectives:**

Postsystolic shortening (PSS) has been proposed as a marker of myocardial dysfunction. Percutaneous transluminal septal myocardial ablation (PTSMA) is an alternative therapy for patients with hypertrophic obstructive cardiomyopathy (HOCM) that results in sustained improvements in atrial structure and function. We investigated the effects of PTSMA on PSS in HOCM patients using speckle tracking imaging.

**Methods:**

Conventional echocardiographic and PSS parameters were obtained in 18 healthy controls and 30 HOCM patients before and half a year after PTSMA.

**Results:**

Compared with the healthy controls, the number of segments having PSS and the average value of PSS were significantly increased in the HOCM patients. At 6 months after PTSMA, both the number of segments having PSS (10.5±2.8 vs. 13.2±2.6; P<0.001) and the average value of PSS (−1.24±0.57 vs. −1.55±0.56; P = 0.009) were significantly reduced. Moreover, the reductions in the average value of PSS correlated well with the reductions in the E-to-Ea ratio (r = 0.705, P<0.001).

**Conclusions:**

Both the number of segments having PSS and the average value of PSS were significantly increased in the HOCM patients. PTSMA has a favourable effect on PSS, which may partly account for the persistent improvement in LV diastolic function in HOCM patients after PTSMA.

## Introduction

Postsystolic shortening (PSS) is considered myocardial shortening after the point of aortic valve closure, which has been known for years. PSS is found in one-third of normal myocardium [1]; however, it is increased by different cardiovascular diseases [2]–[4]. The presence and degree of PSS has been found to be associated with severe cardiac abnormalities [5]. PSS has been suggested as a marker of myocardial ischemia and fibrosis [6]–[7]. A previous study observed that patients with hypertrophic cardiomyopathy (HCM) had more PSS than normal controls [4]. Percutaneous transluminal septal myocardial ablation (PTSMA) is an alternative type of therapy for patients with hypertrophic obstructive cardiomyopathy (HOCM) that may result in the long-term improvement of symptoms and partly reverse myocardial ischemia and fibrosis [8]–[11]. However, it is unclear whether PTSMA can decrease PSS in HOCM patients. Speckle tracking echocardiography (STE) is a novel ultrasonic technique, which can be used for measuring myocardium deformation during the cardiac cycle and is well suited for the quantification of PSS. This study was designed to quantitatively analyse and compare myocardial longitudinal PSS by STE in HOCM patients before and half a year after PTSMA to determine the effect of PTSMA on PSS.

## Methods

This study was reviewed and proved by the Ethics Committee of Beijing Fuwai Hospital. Written informed consents were obtained from all patients and control subjects.

### Study population

The study population consisted of 38 patients with symptomatic obstructive HCM (HOCM) who were referred for PTSMA to our centre between May 2012 and December 2012. However, 5 patients underwent surgical myectomy and mitral-valve replacement, and 3 patients failed to return for the follow-up examination. The final cohort included 30 patients. The diagnoses of HCM were obtained by means of 2-dimensional echocardiography in patients with interventricular septal thicknesses ≥1.5 cm who had no other causes attributed to their left ventricular (LV) hypertrophy. The selection criteria for PTSMA were as follows: the persistence of symptoms despite being administered the maximum tolerated dosage of medication; a left ventricular outflow tract (LVOT) gradient >50 mmHg at rest or >100 mmHg after provocation; accessible septal branches, particularly of the left anterior descending coronary artery; the absence of a significant intrinsic abnormality of the mitral valve; and other conditions for which cardiac surgery was indicated. PTSMA were performed as previously described [12]–[13]. PTSMA success was defined as an improvement in the New York Heart Association (NYHA) class and a reduction in the LVOT pressure gradient of 50% of the baseline. Eighteen age- and gender-matched healthy controls were included from the subjects who visited our hospital for annual routine medical examinations.

### Echocardiography

Each subject underwent an echocardiographic evaluation using a commercially available echocardiographic scanner (IE33, Philips Medical Systems, Best, Netherlands) that was equipped with an S5-1 transducer (frequency transmitted, 1.7 MHz; frequency received, 3.4 MHz) before and half a year after the PTSMA procedure. One lead electrocardiogram was recorded continuously. A 2-dimensionally guided M-mode echocardiography was performed to measure the thicknesses of the interventricular septum and left ventricular posterior wall (LVPW). The wall thickness was measured at the level of the mitral valve and papillary muscles in each of the four myocardial segments and at the apical level in the anterior and posterior segments using parasternal short-axis views. The maximum LV wall thickness was defined as the greatest thickness in any single segment. The left ventricular ejection fraction (LVEF) and the left atrial end-systolic volume (LAV) were calculated using a modified Simpson's biplane method in the apical 4- and 2-chamber views. The maximal early and late diastolic inflow velocities (E and A waves), E-to-A ratio, and deceleration time (DT) of the E wave were obtained using a pulsed-wave Doppler. The sample volume was placed immediately below the level of the mitral leaflet tips in the apical 4-chamber view. The LV outflow tract (LVOT) gradient was measured using a continuous-wave Doppler in the apical 5-chamber view. The TDI of the mitral annulus movement was performed using the apical 4-chamber view. A 1.5-mm sample volume was placed at the lateral side of mitral annular. The velocity of mitral annular was measured in early diastole (Ea) and with atrial contraction (Aa). Analyses of the isovolumic relaxation time (IVRT) were performed.

### Strain Data Acquisition and Analysis

**Figure 1:**
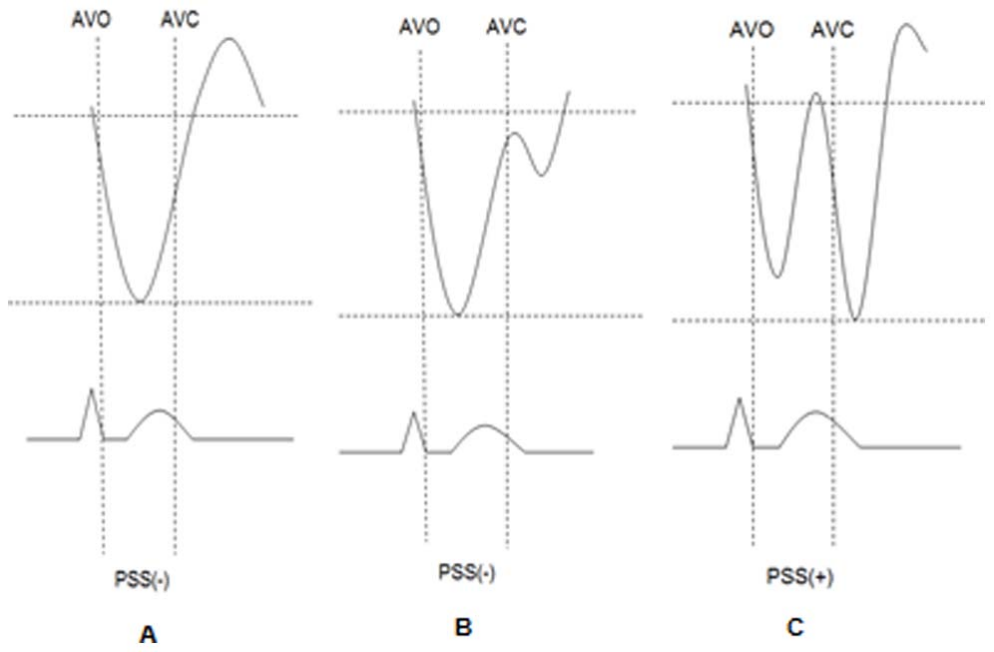
Strain curves during cardiac cycle with (C) and without (A,B) postsystolic shortening (PSS). AVO, timing of aortic valve opening; AVC, timing of aortic valve closure.

Three cardiac cycles were recorded in apical 4-, 2-, and 3-chamber views using grey-scale acquisition at a frame rate over 80 s^−1^. The off-line longitudinal strain data analysis was performed with QLAB 6.0 software (Philips Medical Systems, Andover, Massachusetts, USA). Eighteen segments from 6 LV walls were assessed, namely, the basal, mid, and apical segments for the inferior and anterior septum, and the anterior, anterolateral, inferolateral and inferior wall. The peak negative systolic strain was recorded to assess segmental myocardial systolic function. PSS was defined as the segmental shortening in the diastole beyond the minimum systolic segment length (the peak negative strain in the diastole minus the peak negative strain in the systole). If the minimum segment length was within the systole, PSS was set to zero (Figure 1). The average values of PSS from all 18 segments were then calculated and were considered to be the global PSS.

The reproducibility of measurements of PSS was assessed by inter- and intra-observer agreement. Intra-observer agreement was determined by having one observer repeating the measurements of these variables in all 30 patients. Inter-observer agreement was determined by having a second observer measuring these variables in the same patients. We used the Cohen kappa coefficient to determine inter- and intra-observer agreement of the presence of PSS in different myocardial segments. Spearman, coefficient was performed to assess inter- and intra-observer agreement of average value of PSS in each subjects.

### Statistical analyses

The data are presented as the mean±SD for the continuous variables or as percentages for the categorical variables. The clinical characteristics were compared using the *t*-test for the continuous variables and the chi-square test for the categorical variables. The correlations between changes in the number of myocardial segments with PSS and average values of PSS and the improvement of the E/Ea ratio at half a year after the PTSMA procedure were examined using the Pearson's test. All probability values were for 2-tailed tests. A value of P<0.05 was considered indicative of a statistically significant result. Data processing and statistical analyses were performed using SPSS 17.0 software (SPSS, Chicago, IL, USA).

## Results

The basic clinical characteristics of the HOCM patients and healthy controls are summarised in Table 1. There were no significant differences between the two groups in terms of age, gender, height, weight, heart rate, systolic blood pressure or diastolic blood pressure. Most of the patients experienced severe symptoms of heart failure. The mean NYHA functional class was 2.7±0.3, and 73.3% of the patients were in the NYHA functional class III despite optimal medical therapy that consisted of beta-blockers in 23 (76.7%) patients and calcium-channel blockers in 7 (26.7%) patients. Because of serious side effects or contraindications, 3 patients received neither beta-blockers nor calcium-channel blockers.

**Table 1:** Clinical characteristics of study participants.

	Control	HOCM	P value
	subjects	patients	
	(n = 18)	(n = 30)	
Age,(years)	43.4±11.5	45.6±11.2	0.532
Male, *n* (%)	13 (72.2)	20 (66.7)	0.757
Height,(cm)	166.9±5.5	167.3±5.7	0.833
Weight,(kg)	64.4±8.4	69.3±9.8	0.084
Family history	0 (0)	5 (16.7)	—
of hypertrophic			
cardiomyopathy, *n* (%)			
Previous syncope, *n* (%)	0 (0)	8 (26.7)	—
Previous dyspnea, *n* (%)	0 (0)	20 (64.5)	—
Heart rate, (bpm)	69.1±7.2	69.8±9.4	0.781
SBP, (mmHg)	118.7±6.5	113.3±14.4	0.083
DBP, (mmHg)	70.8±5.6	71.5±9.6	0.783
NYHA classes	—	2.7±0.4	—
β-blocker	0 (0)	23(76.7)	—
Ca channel blocker	0 (0)	8 (26.7)	—

HOCM, hypertrophic obstructive cardiomyopathy; HR, heart rate; SBP, systolic blood pressure; DBP, diastolic blood pressure; NYHA, New York Heart Association.

The conventional echocardiographic parameters in HOCM patients before PTSMA and the control group are shown in Table 2. According to disease characteristics, patients with HOCM exhibited higher LVEF, greater LAV, and thicker LV walls for both the ventricular septum and the posterior wall. The mean baseline resting LVOT gradient for the HOCM patients was 92.4±32.2 mmHg. All of the HOCM patients had abnormal diastolic function before the PTSMA procedure. The transmitral E-wave velocities, A-wave velocities and E/A ratios were similar between the two groups, whereas the DT and IVRT were significantly prolonged in patients with HOCM. The TDI parameters from the lateral mitral annulus showed that the early diastolic peak velocities were significantly lower in HOCM patients. Additionally, the E/Ea ratio in HOCM patients was more than twice as high in healthy controls.

**Table 2:** Conventional echocardiographic characteristics of healthy controls and HOCM patients at baseline and half a year after PTSMA.

Character-	Healthy	HOCM	P value*	HOCM	P value^&^
istics	controls	patients		patients half	
	(n = 18)	at baseline		a year after	
		(n = 30)		PTSMA	
				(n = 30)	
Septum	8.5±1.1	21.3±5.7	<0.001	18.9±6.2	0.122
thickness					
(mm)					
LVPW	8.7±1.5	12.9±3.4	<0.001	12.4±3.2	0.580
thickness					
(mm)					
maxLVT	—	23.6±4.9	—	21.1±5.9	0.080
(mm)					
LAV index	19.1±3.4	44.7±10.7	<0.001	35.3±11.1	<0.001
(ml/m^2^)					
LVEF (%)	63.9±5.5	74.8±8.4	<0.001	74.1±6.0	0.712
LVOT	—	92.4±32.2	—	27.2±17.6	<0.001
gradient					
(mmHg)					
Mitral E	80.7±15.	84.2±23.8	0.582	86.4±20.8	0.711
velocity	9				
(cm/s)					
Mitral A	65.8±21.	83.0±37.4	0.083	90.8±33.9	0.401
velocity	8				
(cm/s)					
E/A ratio	1.4±0.5	1.2±0.5	0.224	1.1±0.5	0.589
DT (ms)	175.2±8.	231.1±46.	<0.001	193.3±18.7	<0.001
	0	4			
IVRT (ms)	76.5±7.0	122.5±22.	<0.001	100?9±18.1	<0.001
		5			
Lateral Ea	12.2±4.0	5.5±1.6	<0.001	7.1±1.3	<0.001
(cm/s)					
Lateral Aa	8.5±2.4	7.9±1.7	0.322	8.1±1.8	0.723
(cm/s)					
E/Ea ratio	7.2±2.4	16.3±6.5	<0.001	12.5±4.2	<0.001

*, P value of comparing between control subjects and HOCM patients at baseline; &, P value of comparing between HOCM patients at baseline and HOCM patients at half a year after PTSMA; HOCM, hypertrophic obstructive cardiomyopathy; LVPW, left ventricular posterior wall; maxLVT,maximal left ventricular thickness;LAV, left atrial volume; LVEF, left ventricular ejection fraction; LVOT, left ventricular outflow tract; DT, E deceleration time; IVRT, isovolumic relaxation time.

**Figure 2:**
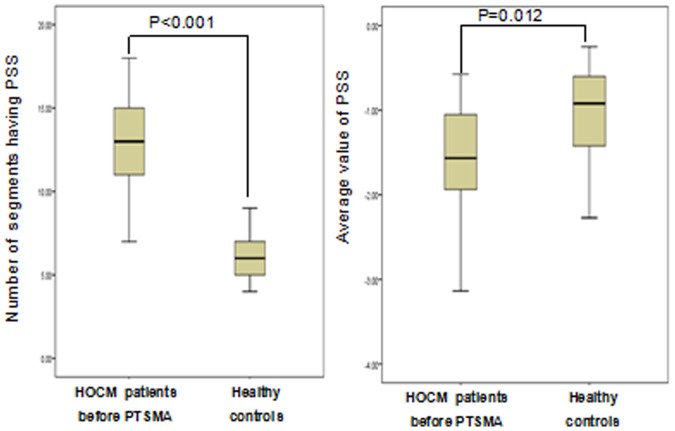
Comparison of the number of segments having PSS (left) and the average value of PSS (right) between obstructive hypertrophic cardiomyopathy (HOCM) patients before septal ablation and healthy controls. PTSMA, percutaneous transluminal septal myocardial ablation.

Figure 2 shows the comparison of the number of myocardial segments having PSS and the average value of PSS between the two groups. As shown, patients with HOCM had a greater number of segments with PSS and greater PSS values than healthy controls(13.20±2.62 vs 5.74±2.05,P<0.001; −1.55±0.57 vs −1.05±0.64,P = 0.012). In HOCM patients group, the inter- and intra-observer agreement is very high in both the presence of PSS in different myocardial segments (Kappa = 0.911,p<0.001;Kappa = 0.831,p<0.001) and the average value of PSS(r = 0.871,p<0.001;r = 0.810,p<0.001).

During the ablation procedure, the mean amount of alcohol that was injected was 2.37±0.90 ml. Right-bundle branch blocks occurred at a rate of 36.7% (11 patients). Transitory trifascicular blocks occurred at a rate of 23.3% (7 patients). No patient underwent a permanent pacemaker implantation following the procedure. There was no peri-interventional mortality during the observational period.

Changes in the conventional echocardiographic parameters in the patients with HOCM at half a year following the PTSMA procedure are shown in Table 2. Patients exhibited an obvious decrease in the LVOT gradient. The PTSMA produced a significant reduction in the interventricular septal thickness. The left ventricular posterior wall also decreased in size, but this change was not statistically significance. The left atrial volume indexed to body surface area was significantly reduced following PTSMA at half a year. There were no significant changes in the E-wave velocity, A-wave velocity, or the E/A ratio. However, the reduction in DT and IVRT were indicative of the amelioration of the LV diastolic dysfunction. Similar trends in the DTI parameters of the diastolic function were also observed. There was an obvious increase in the Ea velocities, resulting in a significant reduction in the E-to-Ea ratio.

**Figure 3:**
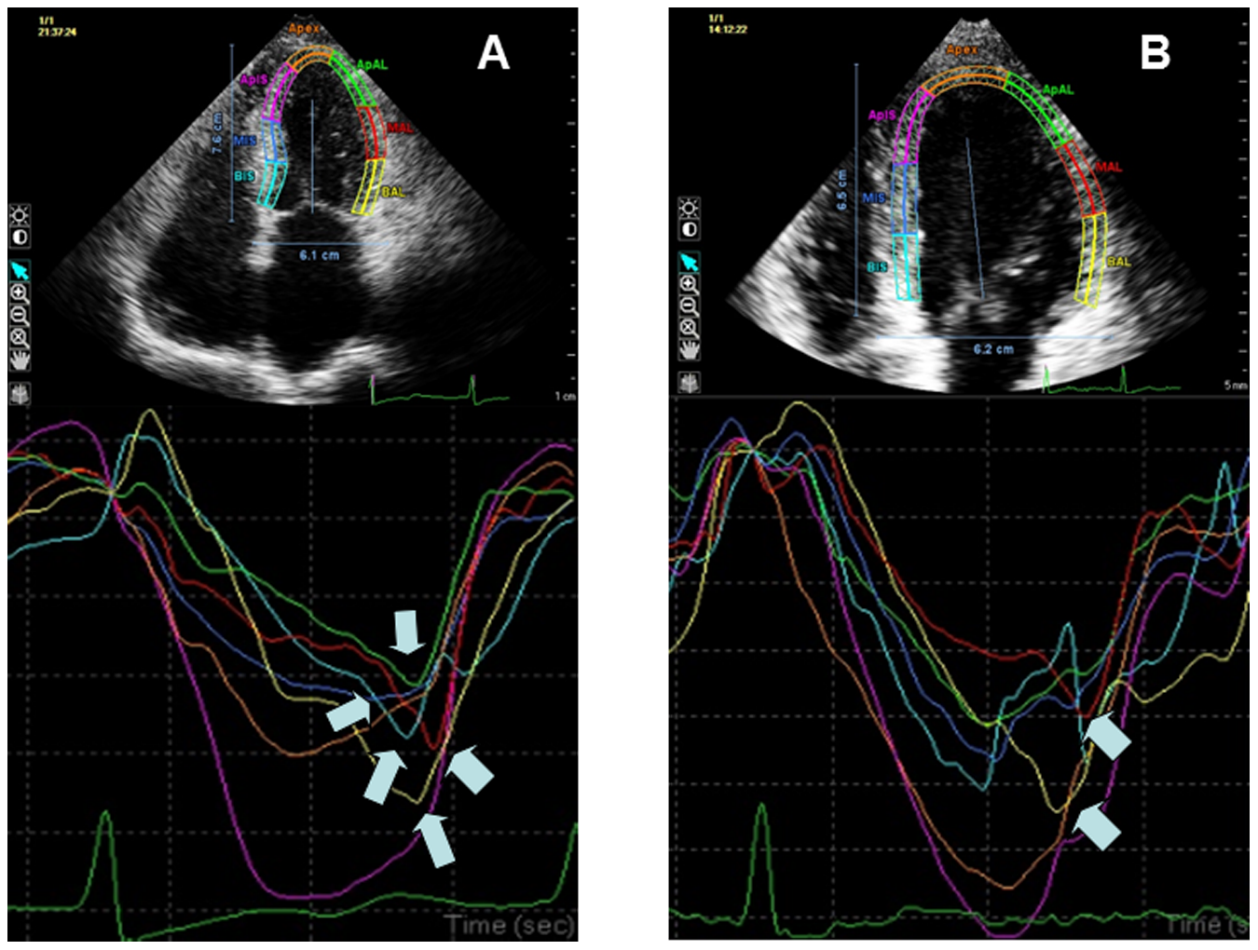
Strain curves from the apical 4-chamber view at baseline (A) and half a year after successful septal ablation (B) in a HOCM patient. The number of segments having PSS was significantly reduced at half a year after PTSMA. ***Arrows***, Strain curves showing postsystolic shortening (PSS).

**Figure 4:**
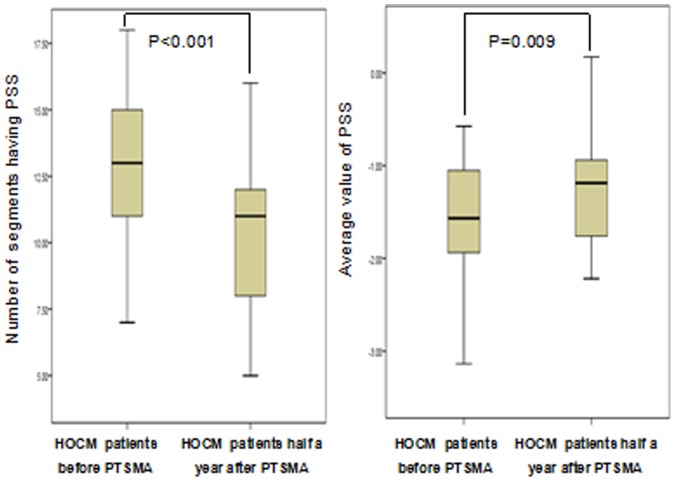
Comparison of the number of segments having PSS (left) and the average value of PSS (right) between HOCM patients before and half a year after septal ablation.

**Figure 5:**
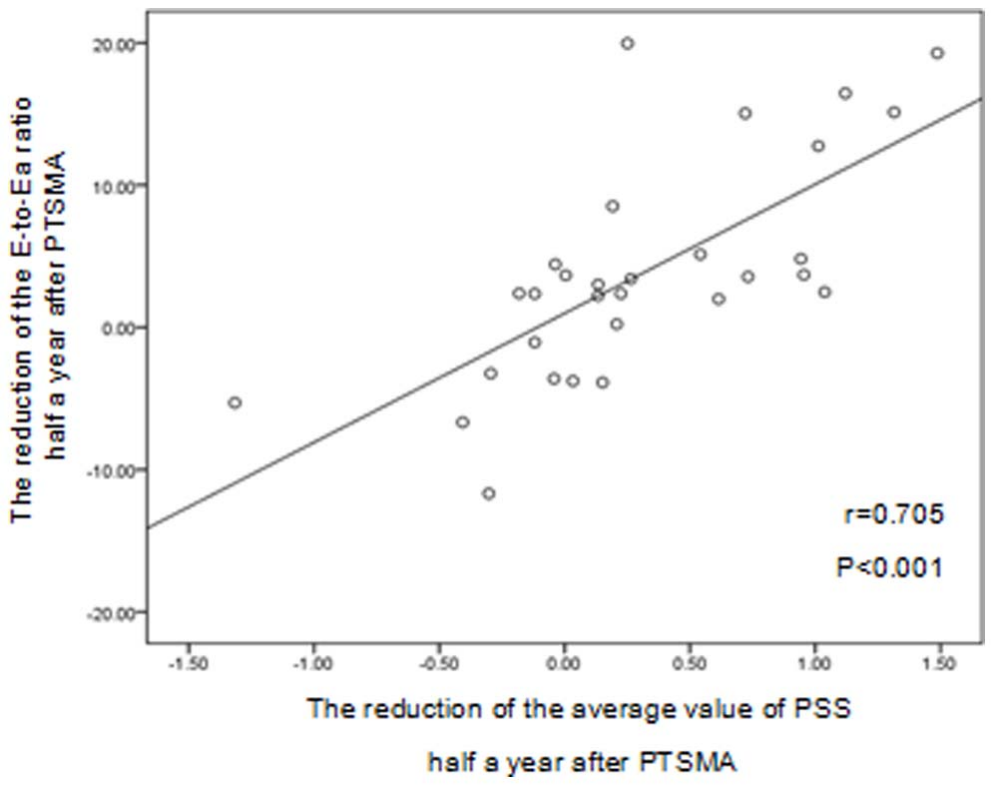
The relationship between the reductions in the average value of PSS and the changes in the E-to-Ea ratio in HOCM patients at half a year after a PTSMA procedure.

As shown in Figures 3, 4 and 5, both the number of myocardial segments having PSS and the average value of PSS were significantly reduced(13.20±2.62 vs 10.53±2.83,P<0.001;−1.55±0.57 vs −1.24±0.57,P = 0.009). In addition, a significant correlation between the reduction of the average value of PSS and the reduction of the E-to-Ea ratio was observed (r = 0.705, P<0.001; Figure 5).

## Discussion

Our study provides unprecedented data regarding the PSS parameters in HOCM patients before and half a year after a PTSMA procedure. We found that compared with healthy controls, both the number of myocardial segments having PSS and the average value of PSS were significantly increased in the HOCM patients. At half a year following PTSMA, both parameters of PSS were significantly improved. Moreover, the reduction of the average value of PSS in the patients was significantly correlated with the reduction of the E-to-Ea ratio.

In 1986, Bertha and colleagues observed myocardial segments shorting after the point of the aortic valve closure into the early diastole using the sonomicrometry technique; they named the phenomenon “postsystolic shortening” [14]. Subsequent studies demonstrated that PSS is a nonspecific feature that occurs in both healthy hearts and in different heart diseases [2]–[4]. However, PSS is observed more frequently and with greater amplitude in the ischemia myocardium than in a normal myocardial [3]. Previous studies showed that PSS could distinguish the ischemia myocardial and reflect the severity of myocardial ischemia [8]–[11]. Myocardial fibrosis is another major reason for an increasing PSS. Plaksej and colleagues observed that with the progression of NYHA functional classes in heart failure patients, the concentration of circulating markers of myocardial fibrosis increased and the PSS index also increased [15]. Tsai further found that in hypertension patients, increased serum procollagen type I carboxyterminal propeptide (PICP), which is considered a circulating maker for myocardial fibrosis, was correlated with increased PSS [16]. Currently, a precise explanation for the association between myocardial fibrosis and PSS is difficult. However, previous studies found that myocardial fibrosis can hinder the unfolding of the left ventricle [17] and delay the myocardial relaxation that may partly contribute to the delayed myocardial shortening into the early diastole.

Hypertrophic cardiomyopathy (HCM) is a genetically transmitted myocardial disease characterised by varying degrees of myocardial hypertrophy. Microvascular dysfunction, characterised by a blunted vasodilator reserve in the absence of an epicardial coronary stenosis, is very common in patients with HCM [18]–[19]. It is mostly a result of the intimal and medial hyperplasia of the intramural coronary arteries and subsequent lumen reduction [20]. Extravascular compression following hypertrophy of the LV wall and the elevated LV end-diastolic pressure is another major reason [21]. Microvascular dysfunction, in turn, can lead to myocardial ischemia because of hypoperfusion in the corresponding area. Myocardial fibrosis is a prominent pathological feature of HCM. Myocardial fibrosis is closely related with the symptoms in HCM patients and is an independent marker of an unfavourable prognosis in this disease [22]–[23]. Thus, it is reasonable to consider whether PSS increases in patients with HCM. However, few studies have focused on PSS in HCM patients. In 2003, Stoylen described PSS in a patient with apical HCM in a case study [24]. In 2006, Ito and colleagues investigated PSS in 30 HCM patients and 30 healthy controls using strain imaging based on tissue Doppler [4]. They found that compared with the healthy controls, the incidence of PSS was noticeably more frequent, and the postsystolic index used to assess the severity of PSS was higher in HCM patients. In our study, we assessed PSS in HCM patients using speckle tracking imaging, which is angle independent and less susceptible to signal noise compared with Doppler strain. However, our results were similar with previous studies. We found that both the number of myocardial segments having PSS and the average value of PSS were significantly increased in the HCM patients.

Currently, PTSMA is considered to be an alternative therapy to surgical myomectomy for patients with HOCM that results in the immediate relief of a LVOT obstruction, the regression of LV hypertrophy and the sustained improvement in LV diastolic function [8]–[9], [25]. Thus, it is reasonable to hypothesise that PTSMA can improve microvascular dysfunction by relieving the extravascular compression in HOCM patients. Recent evidence supports that PTSMA has a favourable effect on microvascular dysfunction. Soliman evaluated the intramyocardial flow dynamics with an adenosine myocardial contrast echocardiography in healthy volunteers and 14 HOCM patients before and 6 months after PTSMA. Soliman found that 6 months after PTSMA, both the myocardial flow reserve and the septal hyperemic endo-to-epi myocardial blood flow ratio were significantly improved [21]. In another study [26], Timmer performed a ^15^O-water PET study to obtain the resting myocardial blood flow and the coronary vasodilator reserve in 15 HOCM patients before and half a year after the PTSMA. In the study, Timmer observed similar results as Soliman, and he further proved that with the improvement of microvascular dysfunction, myocardial energy was restored in the HOCM patients after PTSMA. Furthermore, some studies have suggested that PTSMA can also partly reverse myocardial fibrosis in HOCM patients [11], [27]–[28]. Thus, it is reasonable to consider whether PTSMA can reduce PSS in HOCM patients. However, to our knowledge, there is no study that focuses on the effect of PTSMA on PSS in HOCM patients. Our study showed that PTSMA can significantly reduce PSS in patients with HOCM and may result in improvements in microvascular dysfunction and myocardial fibrosis.

Impaired LV diastolic function is the most common pathophysiological feature of HCM, which has been implicated as the primary determinant of symptoms related to heart failure in HCM patients [29]–[30]. PSS can delay myocardial relaxation resulting in an increased LV filling pressure. Ito reported that the number of segments having PSS correlated significantly with the isovolumic relaxation time in patients with HCM [4]. Ito's results indicated that PSS might contribute to the impaired LV diastolic function in patients with HCM. Significant and sustained improvement in the LV diastolic function has been observed in the short- and long-term following a PTSMA [25]. However, the mechanism of improvement in the LV diastolic function is still unclear. In this study, we found that the decrease in the average value of PSS correlated well with the reduction of the E-to-Ea ratio, which is widely used to estimate the LV filling pressure. Our results indicated that the reduction in PSS might partly account for the sustained improvement of the LV diastolic function in HOCM patients after a successful PTSMA.

This study had several limitations. In addition to the relatively small sample size and the retrospective study design, the invasive measurements of the LV diastolic function were not performed during the course of this study. Instead, we used the E-to-Ea ratio to reflect the LV filling pressure. However, Geske reported that there was only a modest correlation between the estimated LV filling pressure with the use of the E-to-Ea ratio and the directly measured pressure in HCM patients [31].

## Conclusions

In conclusion, compared with the healthy controls, the number of segments having PSS and the average value of PSS were significantly increased in the HOCM patients prior to the septal ablation. PTSMA was found to have a favourable effect on PSS, which may partly account for the persistent improvement in the LV diastolic function in HOCM patients after PTSMA. A larger, prospective study with invasive catheters to evaluate the LV filling pressure is necessary to confirm our results.
